# Fingerphoto morphing attack generation using texture descriptors based landmarks

**DOI:** 10.1038/s41598-024-66790-8

**Published:** 2024-07-13

**Authors:** Hailin Li, Raghavendra Ramachandra

**Affiliations:** Norwegian University of Science and Technology (NTNU), 2815 Gjovik, Norway

**Keywords:** Computer science, Information technology

## Abstract

Smartphone-based biometric authentication has been widely used in various applications. Among several biometric characteristics, fingerphoto biometrics captured from smartphones are gaining popularity owing to their usability, scalability across different smartphones, and reliable verification. However, fingerphoto verification systems are vulnerable to both direct and indirect attacks. In this work, we propose a novel method to generate morphing attacks on fingerphoto biometrics captured using smartphones. We introduce three different image-level fingerphoto morphing attack generation algorithms that can generate high-quality fingerphoto morphing images with minimum distortions. Extensive experiments were conducted on two datasets captured using different smartphones under various environmental conditions. The results demonstrate that the proposed morphing algorithms are highly vulnerable to commercial off-the-shelf and block-directional fingerprint verification systems. To effectively detect morphing attacks on fingerphoto biometrics, we propose the use of fingerphoto morphing attack detection algorithms that utilize both handcrafted and deep features. However, our detection results showed a high error rate in accurately detecting these types of attacks.

## Introduction

Over the past decade, biometric technology has gained widespread acceptance for a variety of security applications owing to its convenience and dependability. It has been utilized in numerous contexts including border control, smartphone access, and individual profiling. Biometric-based user authentication eliminates the need for users to remember multiple passwords for different applications, allowing them to access applications based on their unique physiological and behavioral characteristics. Physiological biometrics, such as the face, fingerprints, and iris, are commonly used in security-related applications.

With the proliferation of technologies and applications, biometric-based user verification has been widely utilized in smartphone applications^1^. Physiological biometrics, such as face, iris, and fingerprints (both contact and contactless), are commonly employed in smartphone devices for authentication purposes. However, the reliable use of biometrics requires a dedicated biometric capture device integrated into a smartphone. The incorporation of such hardware incurs additional costs and restricts its scalability. Consequently, biometric researchers have explored contactless physiological biometric traits acquired using built-in cameras that are available on smartphones^2^. The evolution of smartphones has paved the way for remote verification of user identity for several applications. The high-resolution cameras available in modern smartphones can be widely used by several biometric companies, such as Telos^3^, which has introduced fingerphoto-based user verification systems that are widely used for remote verification. Furthermore, the use of smartphones as sensors for remote verification overcomes the need for a dedicated fingerprint sensor, thus escalating the use of remote verification systems. Fingerphotos are among the most widely studied biometric characteristics for user authentication on smartphones^4^^5^^6^, were captured using a smartphone and processed (post-processing tools that are used for natural photos) using built-in software. This technique is referred to as fingerphoto biometric. The widespread adoption of fingerphoto biometrics has led vendors to develop commercial solutions^7^.

Fingerphoto verification techniques have been extensively studied^4^. The contactless capture of fingerphotos enables distortion-free and hygienic data capture, resulting in a reliable biometric identifier. The wide use of fingerphoto biometrics has raised concerns regarding attacks that can be performed directly and indirectly. Direct attacks describe the attacks direct happens on a biometric capture scanner. This attack requires no knowledge of the biometric system itself. However, an indirect attack involves gaining access to a component of the system and providing forged data at some internal step of authentication. These attackers need to have precise information of the system in order to execute an indirect attack^8^. Direct attacks on fingerphotos have evolved into successful attacks because it is easy to generate fingerphoto artifacts and present them to the capture device. An attacker does not need to know the functional aspects of the fingerphoto biometric system, which is essential for indirect attacks.

Existing studies on fingerphoto attack detection pertain to a presentation attack in which fingerphoto artifacts are presented directly to the capture sensor. Early work in fingerphoto Presentation Attack Detection (PAD) was based on analyzing specular reflection^9^. The back illumination of the smartphone was used to analyze the specular reflection while capturing the fingerphoto for verification. Because the artifact material has different reflective properties compared to natural skin, specular reflection indicates improved detection accuracy. However, the results are limited to a very small proprietary dataset with low-quality artifacts. Fingerphoto presentation attack detection was extensively studied in^10^ by introducing a new dataset with 64 data subjects capturing the right index and right middle finger, resulting in 128 unique identities with photo and print attacks. Fingerphoto PAD algorithms based on Local Binary Patterns (LBP), DSFIT and Locally Uniform Comparison Image Descriptor (LUCID) descriptors were studied, indicating improved attack detection performance. The use of Frangi Filters was studied in^11^ on detecting the attacks on fingerphoto biometrics. A database of 50 data subjects was collected using an iPhone6S, and artifacts were generated using high-quality print and display attacks. The attack detection performance is presented using three different descriptors, LBP, HOG, and BSIF, on the multi-scale representation of the fingerphoto using Frangi Filters. The use of deep features for fingerphoto attack detection is presented in^12^. Two different pre-trained deep CNN, namely ResNet50 and AlexNet, were employed to detect print and display fingerphoto artifacts. A comprehensive study on deep features computed from several pre-trained deep CNN, including AlexNet, ResNet18, ResNet34, DenseNet 121, DenseNet201, and MobileNet, was presented in^13^. The deep features extracted from the color spaces are used to detect fingerphoto presentation attacks. The results presented for print and display attacks indicate the improved performance of ResNet34. Recently, a new fingerphoto presentation detection dataset was published in^14^ and the authors trained the DenseNet 121^15^ and NAS-Net^16^ models to detect the presentation attack. Based on the dataset, another work^17^ evaluated eight different deep learning models to explore the generalization ability of the models. More existing work of contactless fingerprint presentation attack detection can be found in^18^.

Fingerprint verification systems have recently been subject to a novel form of attack, known as morphing attacks. These attacks involve the combination of two or more fingerprint images to create a composite fingerprint image that exhibits the geometric and textural patterns of the source images. Consequently, the morphed images possess a dual identity and correspond to the source fingerprint images utilized in the generation of the morphing image. *Therefore, this paper focus on both generating and detection of morphing attacks on fingerphoto verification system.* The idea of creating a double-identity fingerprint through a morphing process was first introduced by Ferrara et al.^19^. In^19^, two approaches were proposed to combine fingerprints: an image-level approach that directly blends the portion of fingerprints and a feature-level approach that applies an optimal cut-line of two contributing fingerprints based on local orientations, frequencies, and minutia. Another study^20^ proposed a GAN-based approach by combining two latent vectors (corresponding to two different fingerprints) and generating a realistic morphed fingerprint image by feeding the combined latent vectors to the generator. However, the GAN-based approach has a significant drawback in terms of rendering high-quality morphed fingerprints, and therefore exhibits a lower attack potential.

Our work aims to address contactless fingerphoto biometrics captured using a smartphone’s built-in camera, as opposed to existing works that focus on contact-based fingerprints. To the best of our knowledge, our work is the first to address morphing attacks on fingerphoto biometrics. We propose three different algorithms to perform fingerphoto morphing. The proposed method involves image-level blending of fingerphotos from two or more unique data subjects to generate a single fingerphoto morphing image. If this morphed fingerphoto sample is enrolled in the authentication system, it can provide access to all the contributed data subjects, making it vulnerable to evolving attacks, such as injection attacks. These attacks can bypass the smartphone camera and allow the attacker to enrol in morphed fingerphoto biometrics, thereby gaining continuous access to the smartphone. In addition, fingermorphing attacks can be used to target a central biometric database by replacing the enrolled sample. To systematically understand the impact of morphing on smartphone-based fingerphoto biometrics, we introduced the following critical questions:

- Does the fingerphoto morphing image generated using proposed fingerphoto morphing techniques can circumvent the Commercial-off-the-shelf (COTS)^21^ and Block-Directional Fingerprint Verification (BDFV)^22^ systems?
- Does the type of the smartphone device or built-in smartphone camera influence the vulnerability of the fingerprint systems?
- Does the proposed fingerphoto morphing detection techniques can potentially detect the generated attacks?

We address the above research questions through a series of contributions outlined below:

- Proposed a novel method to generate fingerphoto morphing attacks using local descriptors. Three different local descriptors were independently employed to generate fingerphoto morphing attack samples.
- Extensive analysis on benchmarking the vulnerability of the proposed fingerphoto morphing method using Commercial-off-the-shelf (COTS)^21^ based on Verifinger and Block-Directional Fingerprint Verification (BDFV) system^22^.
- Extensive experiments are carried out on the two different fingerphoto datasets. Dataset-I was collected using an iPhone6S in indoor conditions and comprised 100 data subjects, resulting in 378 unique identities. Dataset-II is collected from the publicly available dataset IIITD^6^, the fingerphoto were captured using iPhone5s at 8M resolution, resulting in 37 subjects and 58 unique fingers after manual selection in outdoor conditions.
- Extensive experiments on benchmarking the fingerphoto morphing attack detection using hand-crafted and deep features.

## Proposed method

**Figure 1:**
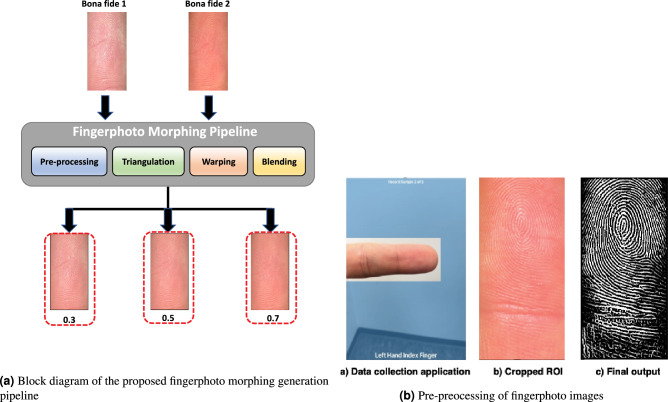
Pipeline of the proposed fingerphoto morphing attack generation and the preprocessing.

This section presents the proposed method for generating finger morphing attacks that can be used as digital attacks in contactless fingerphoto systems. The proposed method was inspired by the conventional face morphing generation method^23^ based on warping and blending. Therefore, the crucial part is the Region Of Interest (ROI) extraction and localization of keypoints to perform warping and blending. Figure 1a shows a block diagram of the proposed method, which has four functional blocks: (1) preprocessing, (2) triangulation, (3)warping, and (4) blending, as discussed in the following sections.

### Pre-processing: segmentation and ROI extraction

In this work, we utilized a fingerphoto dataset obtained through smartphone capture under various environmental conditions^11^. As such, the captured fingerphoto images were expected to exhibit diverse variations owing to the different data capture environments. The fingerphoto samples were collected using a custom smartphone application that featured a rectangular box (ROI) designed to display the finger^11^. To perform alignment and precise cropping of the fingerprint region, a series of steps was followed, including background cropping using color space and clustering, alignment, and enhancement using Frangi filters, as outlined in^11^. Figure 1b depicts the preprocessing steps employed in this study. Ultimately, the preprocessed fingerprint images were resized to $240 \times 480$ pixels and utilized to generate fingerphoto morphing.

**Figure 2:**
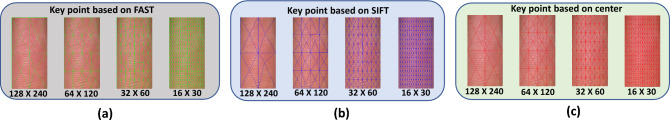
Illustration of triangulation by estimating the point *P* using (**a**) FAST (Algorithm-1) (**b**) SIFT (Algorithm-2) (**c**) Center with different scales (Algorithm-3).

### Triangulation

Triangulation, which is an essential step in generating reliable and robust morphs, relies heavily on identifying landmarks in fingerphoto images. However, detecting landmarks is a challenging task because of several factors, including the low visibility of fingerprint patterns, limited information, separation between ridges and valleys resulting from contactless capture, and difficulty in extracting orientation and minutiae features due to low-quality capture. To address these challenges, we developed a triangulation method that involves dividing the fingerphoto images into grids and estimating keypoints within each grid to construct triangles. Because the keypoints were estimated within rectangular grids on the preprocessed fingerphotos, we believe that they are sufficiently robust to achieve reliable morph generation.

Given the preprocessed fingerphoto *X*, we divide the fingerphoto region into rectangular grids of varying sizes. The size of the grids plays a critical role in achieving reliable fingerprint morphing, as it determines the texture’s coarseness or fineness. Smaller grid sizes result in less texture information, while larger sizes contain more texture information. In this work, we employ a range of grid sizes, including (a) $128 \times 240$ pixels, dividing the fingerphoto into two regions, (b) $64 \times 120$ pixels, dividing it into four regions, (c) $32 \times 60$ pixels, dividing it into eight regions, and (d) $16 \times 30$ pixels, dividing it into 16 regions.

Next, we must identify the point *P* that will serve as the basis for triangulation. To accomplish this, we investigate three methods for optimally positioning point *P* within each rectangular grid: (a) selecting the central point as *P* within the rectangular window, (b) employing a corner detector to identify the corner point and choosing the corner point closest to the center as point *P*, and (c) using a keypoint detector and positioning the keypoint closest to the center of the grid as point *P*. In this study, we employ the Accelerated Segment Test (FAST)^24^ and the Scale-Invariant Feature Transform (SIFT)^25^ to extract corner and key points, respectively. We have also experimented with other keypoint extraction techniques, such as Speeded-Up Robust Features (SURF)^26^ and Binary Robust Independent Elementary Features (BRIEF)^27^, but these two detectors are not able to reliably detect keypoints on fingerphoto images.

Figure 2 depicts the triangulation process for various grid sizes and the methods for localizing keypoint *P*. It is evident that a larger grid size provides more information to perform morphing in the region. The position of key point *P* is of utmost importance in triangulation, and thus, the triangulation process is impacted by the method (FAST, SIFT, and center) employed to compute the keypoint. Additionally, it is important to mention that the methods (FAST, SIFT, and center) used to locate the keypoints exhibit increased sensitivity with smaller grid sizes.

**Figure 3:**
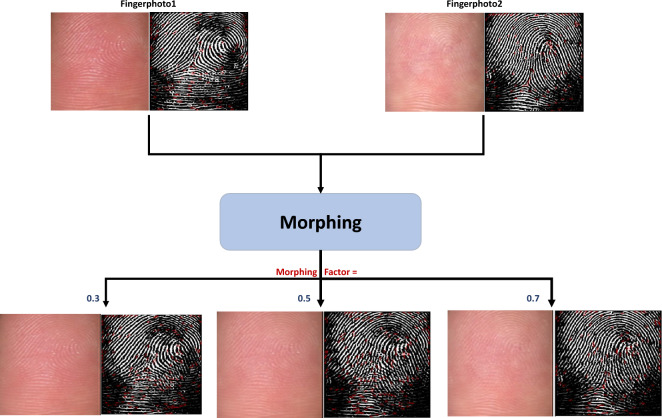
Illustration of fingerphoto morphing images with different morphing factors. Finger photo images are resized to obtain the minutiae points using BDFV^22^, with center based keypoint extraction with a rectangular grid of size $32 \times 60$ pixels. The red color points in the morphing fingerphoto indicates the minutiae points.

### Warping and blending

In the next step, we perform warping and blending operations to generate the final morphed fingerphoto image based on the mapping of the triangles obtained, as discussed above. Given fingerphoto images *x* and *y*, the triangulation process results in $T_{x}$ and $T_{y}$ triangles, respectively. To determine the locations of these triangles in the morphed image $I_{M}$, we select triangle $T_{x1}$ from image *x* and the corresponding triangle $T_{M1}$ in the morphed image $I_{M}$ and compute the affine transforms to map the three corners of $T_{x1}$ to $T_{M1}$, which is repeated for all triangles and with image *y*. Warping is performed by transforming all pixels inside each triangle of image *x* with $I_{M}$ and the same procedure is repeated for image *y*. Finally, the blending operation is performed using Equation 1 to obtain the final morphing image.

1 $$\begin{aligned} I_M(x_{m},y_{m}) = (1-\alpha )T_{x}(x_{i},y_{j}) + \alpha T_{y}(x_{i},y_{j}) \end{aligned}$$

where $T_{M1}$ is the morphed triangle, $x_{w}$ and $y_{w}$ are the two corresponding warped triangles, and $\alpha$ is the blending factor. Initially, $\alpha$ is set as 0.5 that the morph image gets equal contribute from the constituent fingerphotos.

Figure 3 illustrates the fingerphoto morphing images generated based on the center-based keypoint with a rectangular grid of size $32 \times 60$ pixels. Figure 3 also shows the minutiae detection computed from BDFV^22^ on both the bona fide and morphing fingerphoto samples. It is interesting to note that (a) the proposed morphing method can blend the valley and ridges with less distortion and (b) the morphing factor plays a key role in generating the morphing. It can be observed from Fig. 3 that when the morphing factor is 0.3, the generated morphing fingerphoto shows a high resemblance to bona fide 1. A similar observation was noted with a morphing factor of 0.7, which indicates more resemblance with bona fide 2. (c) With a morphing factor of 0.5, the generated fingerphoto resembles both bona fide images 1 and 2. In general, spurious minutiae points are noted more frequently with a morphing factor of 0.5, which can be attributed to the (a) contactless capture of the fingerphoto, which normally results in a low-quality image, and (b) the morphing operation is performed at the image level.

**Figure 4:**
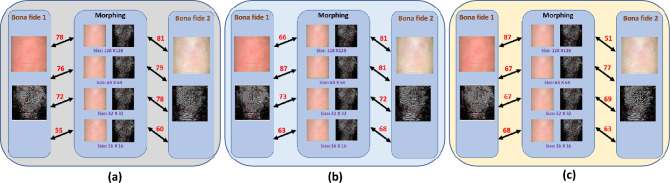
Comparison scores computed using BDFV^22^ on the morphed images generated using (**a**) FAST (Algorithm-1) (**b**) SIFT (Algorithm-2) (**c**) Center with different scales (Algorithm-3). The value upon the arrows indicates the matching scores obtained from the verification application.

Figure 4 illustrates the verification scores computed using the VeriFinger SDK^21^ when morphed image is enrolled and contributory data subjects are probed. The comparison scores are illustrated for all three types of finger photo morphing generation proposed in this work. It is interesting to note that (a) the generated morphing image shows high comparison scores to both contributory data subjects. This can be noticed across all three types of fingerphoto generation method. (b) the selection of scale also influences the verification performance. Thus, these illustrations demonstrate the efficacy of the proposed fingerphoto morphing scheme. For simplicity, we label the morphing algorithm based on FAST as Algorithm-1, SIFT as Algorithm-2 and Center as Algorithm-3. Further, we also label the grid size with $128 \times 240$ pixels as G1, $64 \times 120$ pixels as G2, $32 \times 60$ pixels as G3 and $16 \times 30$ pixels as G4. We follow this convention in the rest of the paper.

### Ethical approval

This dataset that support the findings of this study are publicly available which were under license for the current study. The consent was obtained from all individuals in the previous statement that all of images can be used for research purpose. The informed consent was obtained from all subjects and/or their legal guardian(s). We confirm that all methods were carried out in accordance with relevant guidelines and regulations. The experimental protocol was approved by the Norwegian University of Science and Technology (NTNU) and Indraprastha Institute of Information Technology Delhi (IIIT-Delhi) as the datasets are under the license of these two institutions.

## Results

In the following section, we introduce the fingerphoto morphing databases developed using two distinct datasets. Furthermore, we conduct a vulnerability assessment on two different fingerphoto verification methods. Lastly, we discuss some existing attack detection approaches to illustrate the potential risks associated with morphing samples.

### Generation of fingerphoto morphing databases (FPMD)

**Figure 5:**
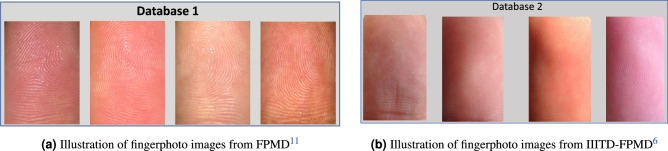
Illustration of fingerphoto images from two different datsets employed in this work.

In this work, we utilized two distinct fingerphoto datasets collected using smartphones under both constrained and unconstrained conditions. As shown in Table 1, the first dataset is from^11^, and comprises 100 data subjects. Fingerphoto images in^11^ are captured using an iPhone 6S with a rear-facing camera. Figure 5a displays example fingerphotos from this dataset. The second dataset is the publicly available IIITD fingerphoto dataset^6^, which consists of 128 classes and over 5100 images under various illumination conditions. The fingerphoto images were captured using an iPhone 5s.

Figure 5b illustrates example fingerphotos from this dataset. We employed the above-mentioned datasets to generate the Fingerphoto Morphing Databases (FPMD) which are described below.

#### NTNU-FPMD

The FPMD is based on the fingerphoto dataset from^28^, which was extended to 100 data subjects. Four fingers (index finger-left, index finger-right, middle finger-left and middle finger-right) were captured for each subject. Eleven images were captured for each finger, resulting in a total of 378 $\times$ 11 = 4158 fingerphoto images. To generate morphing, we used one fingerphoto image, and the rest were used as bona fide samples. To perform morphing, we used only two unique fingers simultaneously to achieve high-quality attacks. The morphing pairs were selected by computing the verification performance^28^ based on BDFV^22^. Thus, for the given finger, we selected the top six fingers with the highest comparison scores. Except for the repetitive pairs, there are 643 morphed samples are generated using one approach. There are 12 algorithms included in total, hence the FPMD dataset contains 643 $\times$ 12 = 7716 morphing images.

#### IIITD-FPMD

IIITD-FPMD^6^ is obtained from a publicly available fingerphoto dataset that consists of three different subsets that address the challenges of environmental illumination, background, and live scans. To ensure that the extracted ROI was of high quality for further experiments, we selected the natural outdoor subsets as a basis, and 37 subjects and 58 different fingers were selected manually with clear texture information. To perform morphing, we followed a procedure similar to that used in the NTNU-FPMD dataset, except that one finger will be morphed with five randomly selected fingers. In total, there were 58 $\times$ 5 $\times$ 12= 3480 morphing images.

**Table 1:** Fingerphoto morphing dataset statistics.

Dataset	Subjects	Unique fingers	Bona fide samples	Morph samples
Dataset1	100	378	3826	7716
Dataset2	37	58	381	3480

### Vulnerability assessment

In this section, we present a quantitative analysis for benchmarking the attack potential of the proposed fingerphoto morphing techniques. The attack potential of the proposed fingerphoto morphing techniques is benchmarked using two different Fingerphoto Verification Systems (FVS): (a) commercial-off-the-shelf (COTS) VeriFinger SDK^21^ and Block-Directional Fingerphoto Verification (BDFV) systems^22^. VeriFinger SDK^21^ is a commercial fingerprint identification software development kit that has fast and reliable fingerprint matching performance. The block directional image is a compact way to represent the structure of a fingerprint and its orientation. As a shape descriptor, the block directional image has been proven to be effective for extracting the macro-characteristics of a fingerprint. We employed these two techniques considering their superior verification performance over other existing SOTA FVS. A detailed evaluation of the five different FVS is discussed in Appendix which forms the basis for this selection. The quantitative value of the vulnerability (or attack potential) is computed by enrolling the morphed fingerphoto (with a morphing factor of 0.5), and comparison scores are obtained by comparing the corresponding fingerphotos from the contributory data subjects. The obtained comparison score was then compared with the preset threshold (e.g., at FAR = 0.1%) to compute the quantitative vulnerability of FVS for morphing fingerphoto samples. In this study, we utilize the vulnerability metrics established in the face morphing community^29^. There are four different metrics that are employed to benchmark the vulnerability (a) Mated Morph Presentation Match Rate (MMPMR)^30^ (b) Fully Mated Morph Presentation Match Rate (FMMPMR)^31^ (c) Morphing Attack Potential (MAP)^32^ and (d) Generalized Morphing Attack Potential (G-MAP)^33^.

In this work, we employ the G-MAP^33^ which is a quantitative measure is used to evaluate the attack potential of morphing images. G-MAP metric was designed to address the limitations of other evaluation metrics, as discussed in^33^.A more detailed discussion about the advantages of G-MAP compared to other metrics is provided in^33^. To compute the G-MAP values, the morphing sample was enrolled in the FRS, and comparison scores were computed by probing the samples of the contributing subjects. If the computed scores exceed the False Acceptance Rate (FAR) threshold, the enrolled sample is considered a successful attack. Therefore, higher values of G-MAP correspond to a higher attack potential for morphing techniques. The G-MAP metric is defined as follows  ^33^:

2 $$\begin{aligned} {\text{G - MAP}} = & \frac{1}{{|{\mathbb{D}}|}}\mathop \sum \limits_{d}^{{|{\mathbb{D}}|}} \frac{1}{{|{\mathbb{P}}|}}\frac{1}{{|{\mathbb{M}}_{d} |}}\min _{{{\mathbb{F}}_{l} }} \sum\limits_{{i,j}}^{{|{\mathbb{P}}|,|{\mathbb{M}}_{d} |}} {\{ \left[ {(S1_{i}^{j} > \tau _{l} ) \wedge \cdots (Sk_{i}^{j} > \tau _{l} )} \right] \times } \\ & \quad \left[ {(1 - FTAR(i,l))} \right]\} \\ \end{aligned}$$

where $\mathbb {P}$ is set of probe images, $\mathbb {F}$ is the set of FRS, $\mathbb {D}$ is the set of Morphing Attack Generation Type, $\mathbb {M}_d$ is the face morphing image set for the Morphing Attack Generation Type *d*, $\tau _l$ indicate the similarity score threshold for FRS (*l*), *FTAR*(*i*, *l*) is the failure to acquire probe image in attempt *i* using FRS (*l*), and || is the number of elements in a set.

**Figure 6:**
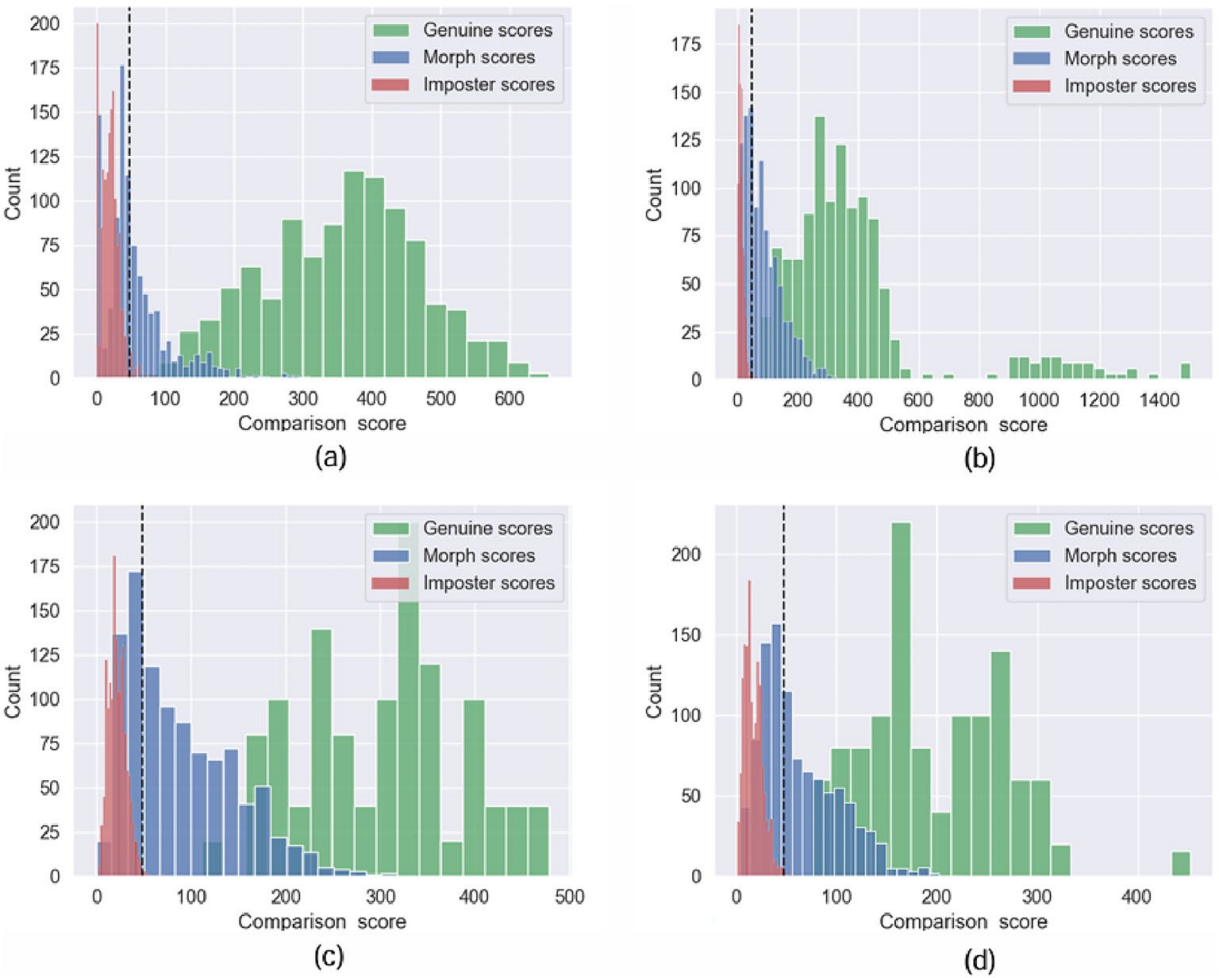
Distribution plot of genuine, zero-effort imposter and morph score computed using COTS^21^ and BDFV^22^ systems. (**a**) Comparison score distribution computed using COTS^21^ on NTNU-FPMD dataset (**b**) Comparison score distribution computed using BDFV^22^ FVS on NTNU-FPMD dataset (**c**) Comparison score distribution computed using COTS^21^ on IIITD-FPMD dataset (**d**) Comparison score distribution computed using BDFV^22^ FVS on IIITD-FPMD dataset. Note that the morphing scores correspond to the Algorithm 3 with G3. The dash line indicate the default matching threshold which is 48 corresponding to FAR = 0.01%.

#### Vulnerability results on NTNU-FPMD dataset

**Table 2:** Morphing vulnerability of BDFV^22^ FVS on NTNU-FPMD dataset in terms of GMAP with multiple probe attempt (GMAP-A)(%) at different FAR (False Accept Rate) thresholds at 0.1%, 1%,10%.

Algorithm / Grid Size	GMAP-A		
	Threshold at FAR =		
	10%	1%	0.1%
Algorithm 1 / G1	86.73	59.65	33.57
Algorithm 2 / G1	79.87	44.19	17.74
Algorithm 3 / G1	90.84	73.74	51.51
Algorithm 1 / G2	75.38	50.26	22.96
Algorithm 2 / G2	72.40	53.26	29.43
Algorithm 3 / G2	92.20	73.79	49.89
Algorithm 1 / G3	74.84	54.95	29.22
Algorithm 2 / G3	76.60	61.41	40.70
Algorithm 3 / G3	92.48	72.91	50.63
Algorithm 1 / G4	75.72	59.75	38.95
Algorithm 2 / G4	76.58	62.77	42.63
Algorithm 3 / G4	92.82	71.77	47.07

**Table 3:** Morphing vulnerability of COTS^21^ on NTNU-FPMD dataset in terms of GMAP with multiple probe attempt (GMAP-A(%)) at different FAR (False Accept Rate) threshold at 0.1%, 1%, 10%.

Algorithm / Grid Size	GMAP-A		
	Threshold at FAR =		
	10%	1%	0.1%
Algorithm 1 / G1	87.45	69.67	33.79
Algorithm 2 / G1	82.31	60.99	23.86
Algorithm 3 / G1	82.02	69.06	36.40
Algorithm 1 / G2	83.41	67.09	28.15
Algorithm 2 / G2	87.40	67.74	28.68
Algorithm 3 / G2	83.39	73.72	45.23
Algorithm 1 / G3	75.75	63.17	30.74
Algorithm 2 / G3	81.82	70.72	40.40
Algorithm 3 / G3	83.75	74.23	46.30
Algorithm 1 / G4	80.54	69.90	41.46
Algorithm 2 / G4	82.31	72.41	44.93
Algorithm 3 / G4	83.06	73.50	46.05

Tables 2 and 3 show the GMAP-A computed based on the comparison scores obtained from two different FVS, including COTS^21^ and BDFV^22^ respectively. The quantitative values of GMAP-A are benchmarked for the three proposed algorithms with four different grid sizes and three different operating thresholds with FAR = $10\%, 1\%$and $0.1\%$. The main observations are as follows:

**Figure 7:**
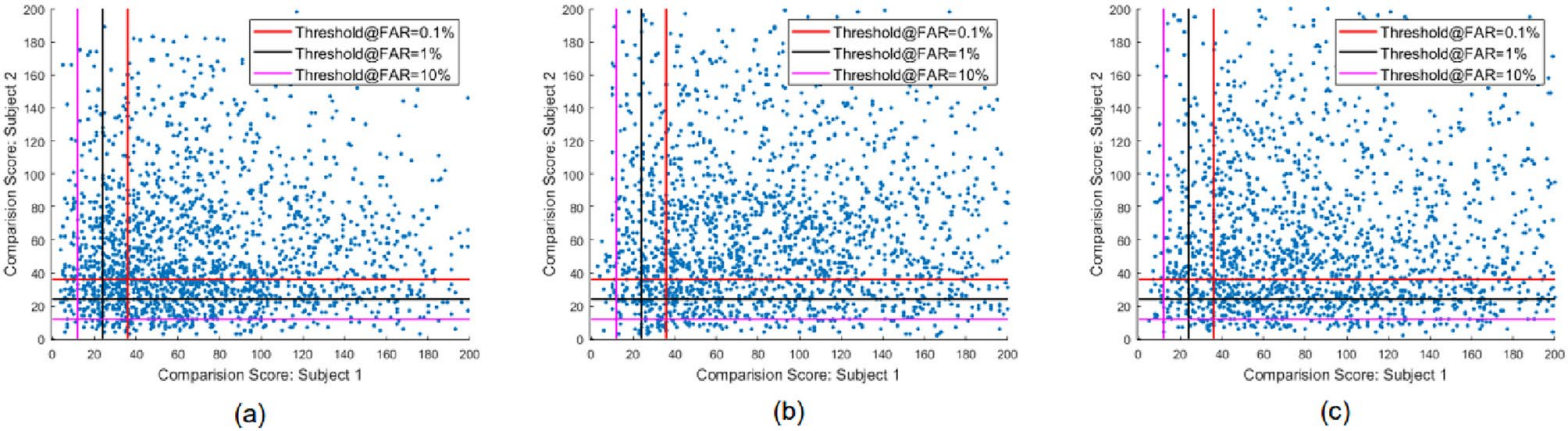
Scatter plot illustrating the comparison score distribution computed on BDFV^22^ FVS on the morphing image generated using (**a**) Algorithm 1/G3 (**b**) Algorithm 2/G3 (**c**) Algorithm 3/G3 on NTNU-FPMD dataset.

- The grid size influences the attack potential of the proposed fingerphoto morphing algorithms irrespective of the FVS.
- Among the four different grid sizes, grid size G1 indicates the best results with Algorithm 1 on COTS^21^ whereas grid size G3 indicates the best results with Algorithm 3 on BDFV^22^ at a lower FAR = $0.1\%$.
- Among the two FVS, the COTS^21^ indicates a higher vulnerability when compared with BDFV^22^. To better interpret the higher vulnerability of the COTS^21^, we conducted a verification experiment on the NTNU-FPMD dataset. Figure 6a shows the distribution of the verification performance of COTS^21^ computed by enrolling one sample per data subject and probing one fingerphoto per subject. The distribution plot corresponded to 400 genuine scores, 500 randomly sampled zero-effort imposters, and 500 randomly sampled morphed scores. For simplicity, we used 500 samples for both zero-effort impostors and morph scores, and we used the morphing finger image generated using Algorithm 3/G3. Similarly, Fig. 6b shows the distribution of the verification performance of BDFV^22^ FVS on the FPMD dataset. It can be noted that the distributions of genuine and zero-effort impostors computed using COTS^21^ are separated compared with BDFV^22^ FVS. Furthermore, the morphing scores are aligned between the genuine and zero-effort impostor score distributions. Thus, the higher vulnerability of the COTS^21^ can be attributed to its accurate verification performance. From our verification experiment, we found that the COTS^21^ indicates a higher verification accuracy (with EER = $0\%$) than the BDFV^22^ FVS (with EER = $0.3\%$) on the NTNU-FPMD dataset.
- In general, at lower values of FAR = $0.1\%$, Algorithm 3/G3 exhibits the best performance with GMAP-A = 46.30 % with COTS^21^ FVS. Algorithm 3/G1 exhibits the best performance with GMAP-A = 51.51% with BDFV^22^ FVS.

**Figure 8:**
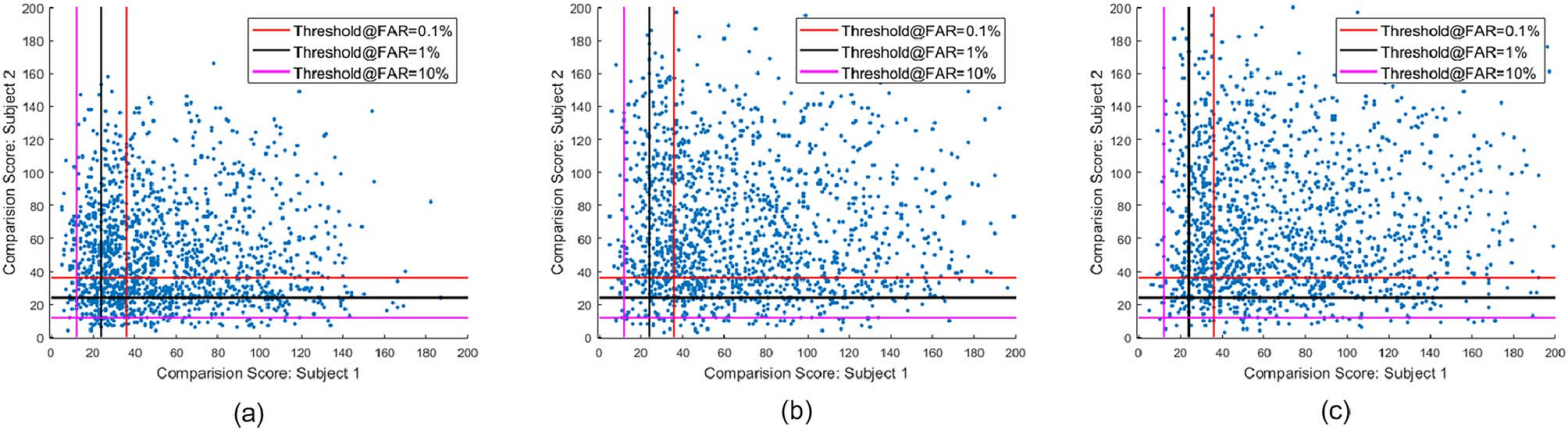
Scatter plot illustrating the comparison score distribution computed on BDFV^22^ FVS on the morphing image generated using (**a**) Algorithm 1/G3 (**b**) Algorithm 2/G3 (**c**) Algorithm 3/G3 on IIITD-FPMD dataset.

Figure 7 shows a scatter plot of the comparison scores from BDFV^22^ FVS for three different operating thresholds: FAR = 10%, 1%, and 0.1%. For simplicity, the plots are shown for Algorithm 1/G3, Algorithm 2/G3 and Algorithm 3/G3. For the system to be vulnerable, the density of the comparison scores must be concentrated in the top-right corner of the plot. As shown in Fig. 7, Algorithms 2 and 3 indicate a higher concentration of scores than Algorithm 1. Specifically, Algorithm 1/G3, Algorithm 2/G3 and Algorithm 3/G3 have 584, 723 and 837 samples respectively surpassing the threshold to match both subjects at FAR= 0.1%

#### Vulnerability results on IIITD-FPMD dataset

This section discusses the vulnerability results of the proposed fingerphoto morphing algorithms for the IIITD-FPMD dataset^6^. Thus, the vulnerability analysis on the IIITD-FPMD dataset reflects the (a) robustness of the morphing algorithms for changes in the image resolution and (b) robustness of the morphing algorithms to the captured conditions as the data are captured in outdoor conditions.

**Table 4:** Morphing vulnerability of BDFV^22^ FVS on IIITD dataset in terms of GMAP with multiple probe attempt (GMAP-A)(%) at different FAR (False Accept Rate) thresholds at 0.1%, 1%,10%.

Algorithm/Grid size	GMAP-A		
	Threshold at FAR =		
	10%	1%	0.1%
Algorithm 1 / G1	91.74	64.88	30.42
Algorithm 2 / G1	84.05	55.50	25.83
Algorithm 3 / G1	95.45	78.89	50.15
Algorithm 1 / G2	90.50	59.52	25.66
Algorithm 2 / G2	93.98	71.26	40.24
Algorithm 3 / G2	95.45	78.75	50.16
Algorithm 1 / G3	91.69	59.38	22.87
Algorithm 2 / G3	94.33	77.41	45.01
Algorithm 3 / G3	95.50	77.65	46.73
Algorithm 1 / G4	94.02	72.97	41.48
Algorithm 2 / G4	96.01	79.40	47.00
Algorithm 3 / G4	95.78	78.71	48.63

**Table 5:** Morphing vulnerability of COTS^21^ on IIITD dataset in terms of G-MAP with multiple probe attempt (GMAP-A(%)) at different FAR (False Accept Rate) threshold at 0.1%, 1%, 10%.

Algorithm/Grid size	GMAP-A		
	Threshold at FAR =		
	10%	1%	0.1%
Algorithm 1 / G1	98.15	85.08	49.93
Algorithm 2 / G1	98.61	76.12	40.48
Algorithm 3 / G1	99.03	88.84	63.80
Algorithm 1 / G2	98.08	79.03	40.05
Algorithm 2 / G2	98.15	85.16	55.68
Algorithm 3 / G2	97.03	85.34	61.30
Algorithm 1 / G3	98.89	81.27	43.82
Algorithm 2 / G3	98.16	87.77	62.23
Algorithm 3 / G3	98.93	88.63	63.26
Algorithm 1 / G4	98.90	87.69	55.94
Algorithm 2 / G4	99.14	89.22	58.56
Algorithm 3 / G4	99.25	89.12	58.89

Tables 4 and 5 show the quantitative results of the vulnerability of two different FVS corresponding to the three different morphing algorithms. Figure 8 shows the scatter plots of the comparison scores obtained using the COTS^21^ for fingerphoto morphing Algorithms 1, 2, and 3 with G3. Vertical and horizontal lines indicate FAR thresholds at 10%, 1%, and 0.1%, respectively. It can be noted that at a higher value of FAR, the systems indicate a higher vulnerability compared to a lower vulnerability. For simplicity, only case G3 with COTS^21^ is illustrated. In this case, the Algorithm 1/G3, Algorithm 2/G3 and Algorithm 3/G3 have 530, 799 and 827 samples respectively surpassing the threshold to match both subjects at FAR= 0.1%

Tables 4 and 5 shows the vulnerability of the BDFV^22^ and COTS^21^ FVS presented using GMAP-A on the IIITD-FPMD dataset. Based on the obtained results it can be noted that:

- Among two FVS, the COTS^21^ FVS indicates the higher vulnerability on all three different morph generating algorithms with four different grid sizes. The higher vulnerability of COTS^21^ FVS can be attributed to the higher verification performance of COTS^21^ FVS compared to BDFV^22^ FVS, as shown in Fig. 6c,d.
- At lower FAR = 0.1%, Algorithm 3 /G2 indicates the higher vulnerability of BDFV^22^ FVS with GMAP-A = 50.16% and Algorithm 3/G1 indicates a higher vulnerability of the COTS^21^ FVS with GMAP-A = 63.80% .
- The grid size influences the vulnerability of the fingerprint verification system. The grid size G3 indicates a degraded vulnerability performance compared to the other grid sizes.
- The vulnerability is noticed to have a higher value of GMAP-A for both FVS at higher FAR thresholds.

### Fingerphoto morph attack detection (FMAD) assessment

In this section, we discuss fingerphoto morphing-detection techniques. Because fingerphoto morphing is introduced for the first time in this work, we benchmark both handcrafted and deep features to detect fingerphoto morphing. We have evaluated feature extraction techniques such as Local Binary Patterns (LBP)^34^, deep features extracted using AlexNet (from FC6 layer)^35^, ResNet50 (from Avg Pool layer)^36^, EfficientNet-b0^37^ and transformer features from Vision Transformer^38^. The extracted features are classified in the next step using a linear Support Vector Machine (SVM). The quantitative results of the fingerphoto morphing attacks are presented using ISO/IEC metrics, namely the Morphing Attack Classification Error Rate (MACER (%)) and Bona fide Presentation Classification Error Rate (BPCER (%)). MACER indicates the proportion of morphing attacks incorrectly classified as bona fide presentations in a specific scenario and BPCER indicates the proportion of bona fide presentations incorrectly classified as morphing attacks in a specific scenario. In addition, we have also included Detection-Equal Error Rate (D-EER) where MACER = BPCER.

In this work, we benchmark the performance of fingerphoto morphing attack detection using an inter-dataset evaluation protocol. Thus, training and testing were performed on different datasets. Table 6 lists the number of samples in the training and testing partitions on both the NTNU-FPMD and IIITD-FPMD datasets. Given the dataset, we perform 50-50 partitions to obtain training and testing sets based on the unique fingers, and morphing was carried out within the partition to achieve complete disjoint identity sets for training and testing. We used only Algorithm 3/G3 for the morphing samples, as it was the best-performing algorithm for achieving the highest vulnerability with different FVS employed in this work. Table 7 presents the quantitative results of the proposed fingerprint detection. The following are critical observations based on the obtained results.

- Among all feature extraction approaches, the proposed morphing technique shows high potential risk that is hard to detect using either handcraft-based method or deep learning based methods.
- When training with NTNU-FPMD and testing with IIITD-FPMD dataset, LBP and ViT both achieve the best result with D-EER = 13.68%. When training with IIITD-FPMD and testing with NTNU-FPMD dataset, the best result is obtained by ResNet with D-EER = 23.22%.
- Among deep learning-based approaches, ResNet shows high generalization properties that achieve D-EER = 23.80 % when trained with NTNU-FPMD and D-EER = 23.22 % when trained with IIITD-FPMD dataset.
- Training with FPMD and testing with IIITD-FPMD indicates a better average detection performance.
- Comparing handcraft-based methods with deep learning based methods, deep features indicates an advantage over handcrafted features.

**Table 6:** Statistics of Morphing Attack Detection datasets, note that the morphing samples are generated by Algorithm 3/G3.

Dataset	Number of images			
	Bonafide		Morph	
	Train	Test	Train	Test
NTNU-FPMD	1980	1800	348	295
IIITD-FPMD	300	81	232	58

**Table 7:** Detection performance of the proposed Fingerphoto MAD Algorithms on the morphing images generated using Algorithms 3/G3.

Detection method	Training dataset	Testing dataset	BPCER @ MACER=		D-EER (%)
			5(%)	10(%)	
LBP-SVM	NTNU-FPMD	IIITD-FPMD	18.95	13.68	13.68
	IIITD-FPMD	NTNU-FPMD	83.02	68.00	36.22
AlexNet-SVM	NTNU-FPMD	IIITD-FPMD	54.32	50.62	34.51
	IIITD-FPMD	NTNU-FPMD	70.08	60.80	32.19
ResNet-SVM	NTNU-FPMD	IIITD-FPMD	35.80	30.86	23.80
	IIITD-FPMD	NTNU-FPMD	45.15	34.01	23.22
EfficientNet-SVM	NTNU-FPMD	IIITD-FPMD	39.51	29.63	20.84
	IIITD-FPMD	NTNU-FPMD	62.66	47.88	29.29
ViT-SVM	NTNU-FPMD	IIITD-FPMD	18.95	13.68	13.68
	IIITD-FPMD	NTNU-FPMD	89.60	86.68	50.00

## Discussion

Based on the observations from the experiments and obtained results, the research questions formulated in Section are answered below.

- Q1. Does the fingerphoto morphing image generated using proposed fingerphoto morphing techniques can circumvent the Commercial-off-the-shelf (COTS)^21^ and Block-Directional Fingerprint Verification (BDFV)^22^ systems?
  - Based on the quantitative results tabulated in Tables 2, 3, 4 and 5 the fingerphoto samples generated using the proposed fingerphoto morphing algorithms indicate the vulnerability on both Fingerphoto Verification Systems (FVS). The quality of the morphing image depends on the quality of the source fingerphoto images employed to generate morphing. The higher vulnerability observed with IIITD-FPMD can be attributed to the higher quality of the fingerphoto samples. Among the three fingerphoto morphing algorithms, Algorithm-3 indicates the highest vulnerability for both the FVS.
- **Q2**. Does the type of the smartphone device or built-in smartphone camera influence the vulnerability of the fingerprint systems?
  - Based on the experimental results tabulated in Tables 2, 3, 4 and 5 type of the smartphone and capture conditions influence the vulnerability. The higher the quality of the captured source fingerphotos used for morphing, the higher the vulnerability.
- **Q3**. Does the proposed fingerphoto morphing detection techniques can potentially detect the generated attacks?
  - Based on the extensive experiments reported in Table 7, the deep features especially extracted using resNet indicates the best detection accuracy compared with other deep features and hand-crafted features. However, the obtained detection accuracy indicates that feature extraction SVM-based approaches are not robust to capture conditions and image quality.

## Conclusion

Smartphone-based fingerphoto verification has been widely studied and has resulted in several academic and commercial solutions. However, fingerphoto biometrics is vulnerable to attacks that can be performed directly or indirectly on a biometric system. In this work, we introduced a novel attacks on fingerphoto biometric systems based on image-based morphing. We proposed three different algorithms based on corner features to generate a fingerphoto morphing image from the contributory data subjects. The efficacy of the morphing images generated using the proposed method was benchmarked with vulnerability analysis using both commercial and open-source fingerprint verification systems. The obtained results indicate the high attack potential of fingerphoto images generated using the proposed morphing technique. Furthermore, the vulnerability of the morphing images also depends on the quality of the fingerphoto samples used to generate morphing. Among the two FVS employed in this work, the COTS^21^ indicated the highest vulnerability. Furthermore, fingerphoto morphing attack detection algorithms have been introduced based on both handcrafted and deep features. Extensive experiments indicate that the best detection performance is achieved by the visual transformer features inter-dataset experiments.

### Supplementary Information

Supplementary Information.

## Data Availability

The datasets used and analyzed during the current study are available from the corresponding author on reasonable request.
