# Supplementary material for: Fingerphoto morphing attack generation using texture descriptors based landmarks
Source: Sci Rep. 2024 Jul 13;14:16182. doi: 10.1038/s41598-024-66790-8 (PMC11246451; doi:10.1038/s41598-024-66790-8)
Supplement: Supplementary file 1 — Supplementary Information. [file 41598_2024_66790_MOESM1_ESM.pdf]

Appendix

Morphing using different fingerprint pattern

In this section, we performed additional experiments to generate the morphing images from different fingerprint patterns. To this extent, we have employed the proposed Algorithm 3/G3 (for simplicity) to generate the morphing images that are further evaluated for the attack potential using the BDFV[22] fingerphoto verification system. Supplementary Figure 1 shows the different fingerphoto patterns corresponding to bona fide subjects 1 and 2 that are used to generate the morphing images. We have also included the verification score computed using BDFV[22] by comparing the morphing images with the corresponding bona fide images. The obtained verification scores indicate the vulnerability (the comparison scores are greater than 48, which corresponds to the threshold of FAR = 0.01%) of the BDFV[22] system. Therefore, the proposed method is not sensitive to the type of the finger pattern. These results are included in the appendix of the revised paper. Note that, in this work, we select the image pairs for morphing using the BDFV[22] fingerphoto verification systems, as discussed in the manuscript (page number: 6, Section **NTNU-FPMD** and **IIITD-FPMD**).

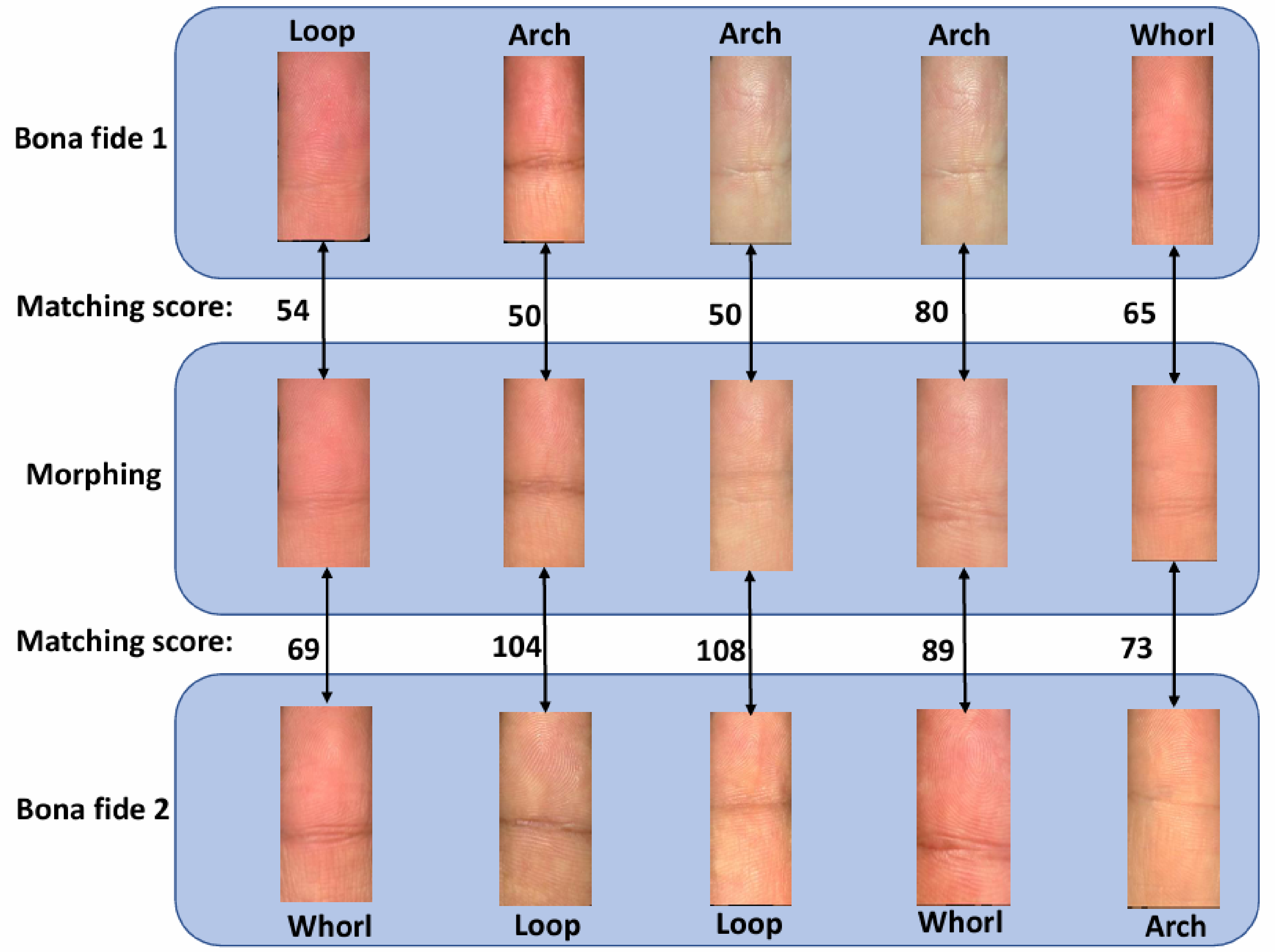

Supplementary Figure 1. Morphing example of the combination of different fingerprint patterns

Morphing using fingerphoto with different intensity

To provide insights into the robustness of the proposed morphing algorithms to varying intensities between the fingerphoto images to be morphed, we performed additional experiments. Supplementary Figure 2 illustrates an example of morphing images generated using Algorithm 3/G3 (for simplicity) when the image pairs to be morphed are of different brightness values. The attack potential of the morphing images was quantified by computing the comparison scores using BDFV[22] fingerphoto verification systems. The obtained scores indicate the vulnerability of the BDFV[22] fingerphoto verification system, as the comparison scores exceeded 48 (corresponding to the FAR = 0.01%). These results indicate that the proposed method is not

sensitive to the brightness between the images that are morphed.

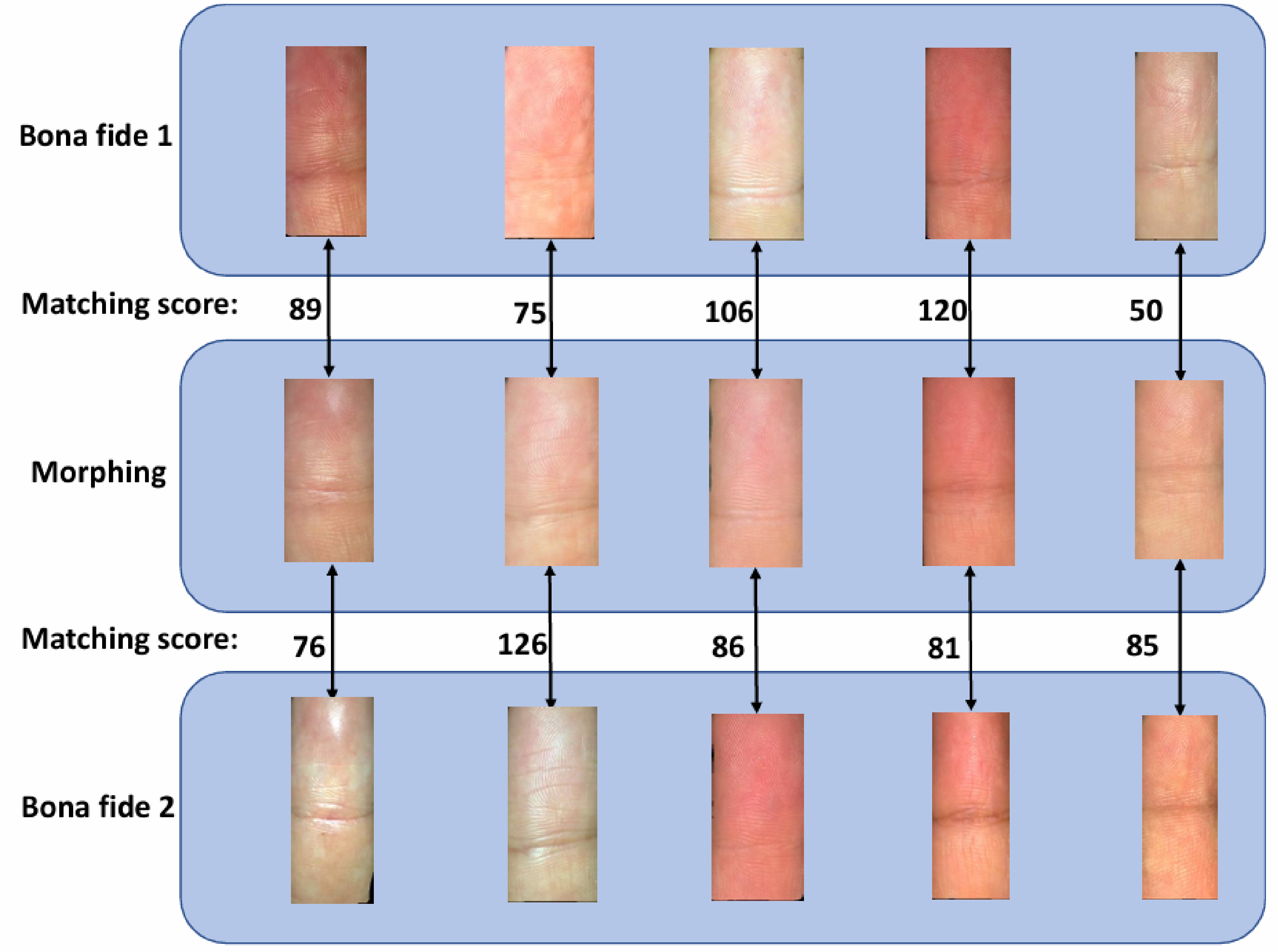

Supplementary Figure 2. Morphing example of the combination of different brightness

Morphing generation from cross dataset identities

In this section, we perform cross-dataset morphing by creating a new morphing dataset using the input samples from different datasets (NTNU-FPMD and IIITD-FPMD). We randomly selected 37 subjects from the NTNU-FPMD dataset and performed one-to-one morphing with the subjects from the IIITD-FPMD dataset. Then we followed the evaluation procedure and obtained the GMAP-A = 59.88%, 29.80% and 18.16% with FAR thresholds at 10%, 1%, and 0.1% using BDFV[22] fingerphoto verification system. These results indicate that, the proposed method is marginally affected by the data variation.
